# The efficacy of non-complete omentectomy in the radical gastrectomy for gastric cancer: a meta-analysis and systematic review

**DOI:** 10.1007/s00423-025-03760-2

**Published:** 2025-06-03

**Authors:** Chenglong Gao, Wenchao Shi, Baoming Zhang, Qiang Tang, Huiyu Chen, Zengtao Bao

**Affiliations:** 1 Department of Gastrointestinal Surgery, Lianyungang Clinical College of Nanjing Medical University, The First People’s Hospital of Lianyungang, Lianyungang, 222016 PR China; 2 Department of Gastrointestinal Surgery, The First People’s Hospital of Lianyungang, Lianyungang, 222016 PR China; 3 The First Affiliated Hospital of Kangda College of Nanjing Medical University, Lianyungang, 222016 PR China; 4 Xuzhou Medical University Affiliated Hospital of Lianyungang, Lianyungang, 222016 PR China

**Keywords:** Gastric cancer, Omentum, Gastrectomy, Omentectomy, Outcomes

## Abstract

**Background:**

Surgery is considered a necessary treatment for gastric cancer (GC), but the extent of resection remains controversial. This study aimed to evaluate the efficacy of non-complete omentectomy (NCO) in GC patients undergoing radical gastrectomy.

**Methods:**

We searched for studies of non-complete omentectomy versus complete omentectomy (CO) published before February 2024 from PubMed, Web of Science, and Cochrane Library databases. From the extracted clinical data, we compared surgical, survival, and recurrence outcomes between the two groups.

**Results:**

Thirteen studies with a total of 4255 patients were included. The meta-analysis showed that compared with the CO group, the NCO group was associated with a lower overall recurrence rate, shorter operative time, and fewer postoperative complications. However, there was no significant difference in the number of harvested lymph nodes and peritoneal recurrence rate between the two groups. The NCO group was associated with the higher 3-year overall survival (OS) rate (RR = 0.95, 95% CI = 0.91–0.99, *P* = 0.02), 5-year OS rate (RR = 0.91, 95% CI = 0.87–0.96, *P* = 0.0006), and 5-year relapse-free survival (RFS) rate (RR = 0.93, 95% CI = 0.87–0.99, *P* = 0.02). However, it was not associated with the 3-year RFS rate (RR = 0.95, 95% CI = 0.89–1.01, *P* = 0.12) compared with the CO group.

**Conclusion:**

Regarding surgical, survival, and recurrence outcomes, performing NCO versus CO during radical gastrectomy provides no significant advantage. However, future high-quality and well-designed randomized controlled trials are necessary to validate the results.

**Supplementary Information:**

The online version contains supplementary material available at 10.1007/s00423-025-03760-2.

## Background

Gastric cancer (GC) has become a great global human health hazard. According to GLOBOCAN 2020, it is the fifth most common malignant tumor and the fourth leading cause of cancer-related death in the world [1]. Meanwhile, GC has become the second most common cancer and the second leading cause of cancer-related deaths in China [2]. Compared with most developed countries, it has a higher incidence rate, mortality, and 5-year prevalence rate [2]. Radical gastrectomy remains the mainstay of treatment for resectable gastric cancer, although the efficacy of adjuvant radiotherapy, immunotherapy, and targeted therapy has been proven today [3].

There is still controversy about the extent of resection in radical cancer surgery. According to the Japanese gastric cancer treatment guidelines 2018 (5 th edition), resection of the greater omentum is recommended during standard gastrectomy for patients with T3 or deeper tumors [4]. For patients with T1/T2 GC, guidelines recommend that the omentum be preserved more than 3 cm from the gastroepiploic artery [4]. Meanwhile, total omentectomy for D1 and D2 gastrectomy is recommended by the Chinese Society of Clinical Oncology (CSCO) [5]. The available studies are mainly retrospective observational studies and the only randomized controlled trial (RCT) that analyzed the effects and outcomes of CO and NCO in radical gastrectomy [6–15]. Due to the significant bias in the abovementioned studies, several meta-analyses have previously been performed to statistically analyze the data in the literature [16–24], illustrating that NCO has similar or even better outcomes than CO in radical gastrectomy.

Based on the results of a recent literature search, we identified three new studies comparing CO and NCO for radical gastrectomy in patients with GC [25–27]. Therefore, our meta-analysis was conducted to update the existing evidence from the most recent study data comparing CO and NCO for outcomes of surgery, survival, and recurrence in radical gastrectomy.

## Methods

This meta-analysis was carried out in accordance with the Preferred Reporting Items for Systematic Reviews and Meta-Analyses (PRISMA) statement [28]. The study was registered on the PROSPERO database (CRD42023449075).

### Search strategy

The PubMed, Web of Science, and Cochrane Library databases were systematically searched to investigate the efficacy of NCO versus CO in gastrectomy for GC. The specific search strategy was as follows:

Pubmed: (“gastric cancer” OR “gastric carcinoma” OR “stomach cancer” OR “gastric adenocarcinoma”) AND (“omentum” OR “omentectomy”) AND “gastrectomy”.

Web of Science: ((TS = (gastric cancer)) AND TS = (omentectomy)) AND TS = (gastrectomy).

Cochrane Library: gastric cancer in Title Abstract Keyword AND omentectomy in Title Abstract Keyword AND gastrectomy in Title Abstract Keyword.

There were no date or language restrictions in the search strategy. The latest recent search was conducted on 26 February 2024.

### Study selection criteria

The inclusion criteria were as follows: [1] studies on patients undergoing radical gastrectomy for GC and comparing the use of CO and NCO during surgery; [2] the literature was required to provide one or more of the indicators of the patients’ 3-year or 5-year overall survival rate and relapse-free survival rate, postoperative complications, and recurrence of the tumor. The exclusion criteria for this study were as follows: [1] studies in the form of abstracts, animal experiments, case reports, letters, protocols, and reviews; [2] studies with incomplete or inaccurate data.

### Data extraction

Two independent reviewers (CG and ZB) selected papers on the basis of title, abstract, and full text. The following data were extracted from the studies that met the criteria: first author, year of publication, study country, study period, study design, population size, characteristics of the study population, surgical approach, surgical procedure, operative time, estimated blood loss, number of harvested lymph nodes, postoperative complications, overall recurrence rate, peritoneal recurrence rate, 3-year and 5-year overall survival (OS) rates, and 3-year and 5-year relapse-free survival (RFS) rates. Conflicts of opinion encountered between authors were resolved by discussion. The results were checked by a third reviewer (WS).

### Risk of bias assessment

For randomized clinical trials (RCTs), we used the Cochrane Risk of Bias tool [29], and for included observational studies, we used the Newcastle Ottawa Scale (NOS) [30]. NOS categorizes each observational study into stars ranging from 0 to 9 based on patient selection, comparability, and outcomes. A score ≥ 7 on the NOS scale indicated high-quality studies. Funnel plot analysis was used to detect publication bias when more than 10 studies were included.

#### Statistical analysis

Our meta-analysis was performed using Review Manager (RevMan) Version 5.4.1 software (Cochrane, London, UK). For dichotomous outcomes, the Mantel‒Haenszel (MH) method was used to calculate risk ratios (RR) and corresponding 95% confidence intervals (CI). In the case of continuous outcomes, mean differences (MD) and corresponding 95% CI were calculated according to the inverse variance method. When the mean or standard deviation (SD) of an endpoint was lacking in the data, calculations were made based on the reported median, range, or interquartile range (IQR), if provided [31, 32].

The heterogeneity of the studies was assessed by means of the *I*^*2*^ statistic. The random-effects model was performed when *I*^*2*^ > 50% indicated significant heterogeneity. Otherwise, the heterogeneity was considered acceptable and the fixed-effects model was used. Statistical significance was set at *P* < 0.05. In addition, subgroup analyses were performed according to different study designs.

## Results

### Search results and study characteristics

**Fig. 1:**
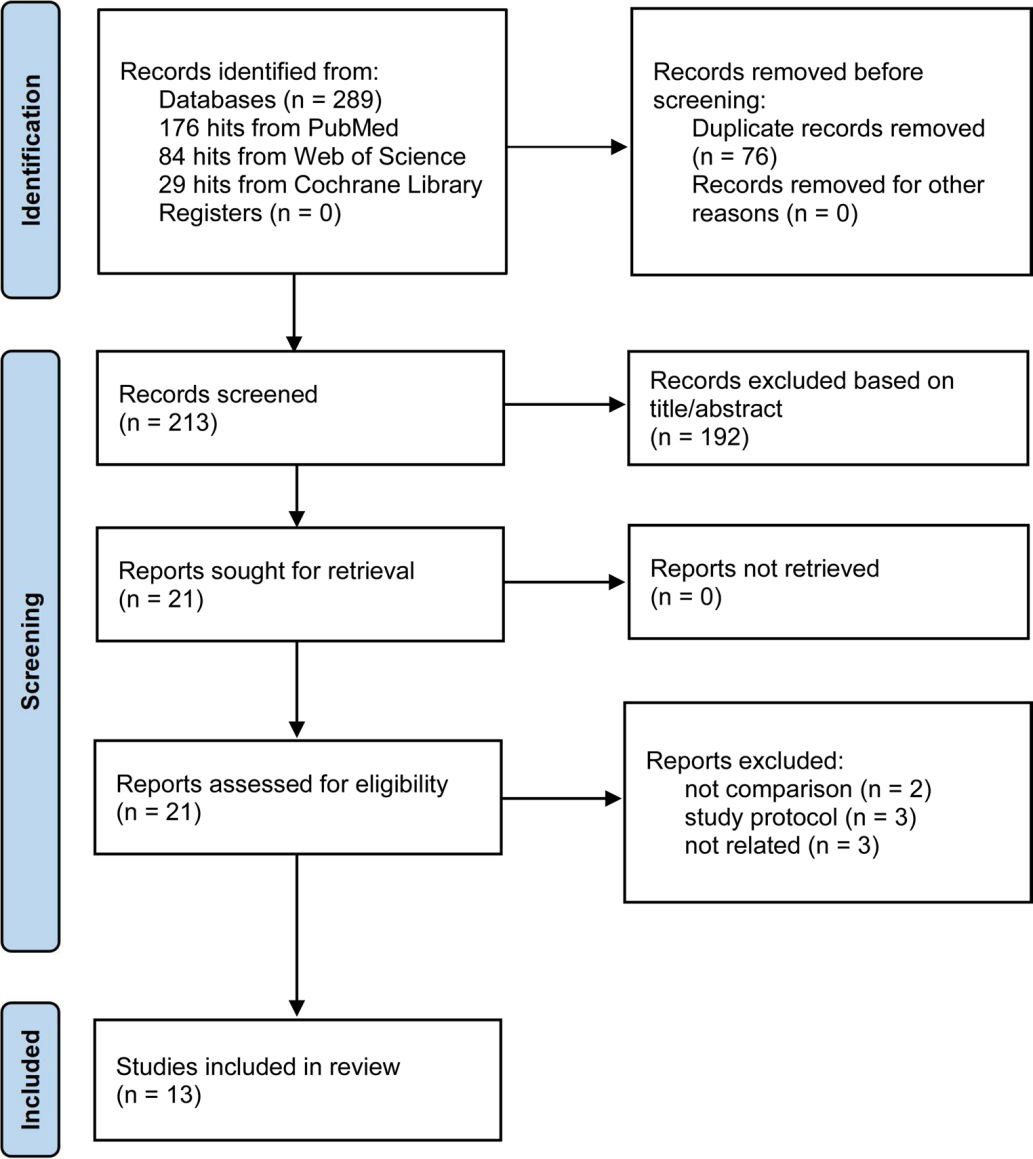
PRISMA flowchart of the literature search and selection procedure

**Fig. 2:**
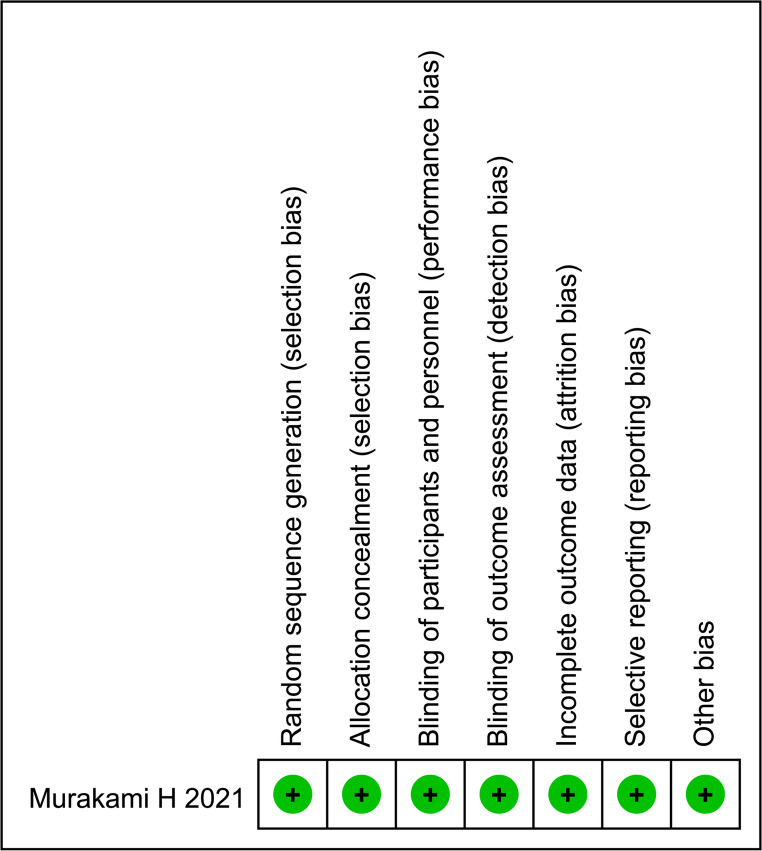
Risk of bias summary for the included RCT studies

From the final literature search, a total of 289 studies (176 in PubMed, 84 in Web of Science, and 29 in the Cochrane Library) were retrieved according to the search strategy. While the search covered 289 studies, 13 studies were included in our meta-analysis after eliminating duplicates and screening the titles, abstracts, and full text of the literature according to strict inclusion and exclusion criteria. The details of the screening are shown in Fig. 1. The total number of patients included in the thirteen studies was 4255 (2382 in the CO group and 1873 in the NCO group). The included studies were published between 2008 and 2023, with a study period ranging from 2000 to 2021, and came from four countries, China, Korea, Japan, and the US. The general information of the included studies, as well as the population characteristics, are summarized in Tables 1 and 2. According to the Cochrane Risk of Bias tool, one RCT study included had a mild risk of bias, as shown in Fig. 2. The remaining 12 studies were retrospective and, according to the Newcastle-Ottawa Scale, all achieved a score of more than 7, thus indicating that they can be regarded as high-quality studies.

**Table 1:** Study general information

Authors/Year	Study design	Study Period	Country	Group	Sample size	Gender (Male/Fmale)	Age (Years), Mean or Median	BMI(kg/m2), Mean or Median	Quality score
Song M al. 2023	Retrospective	2019–2021	China	CO	50	29/21	NA	NA	9
				NCO	58	29/29	NA	NA	
Jeong SA al. 2023	Retrospective PSM	2005–2017	Korea	CO	91	198/112 NA	58.92 ± 18.1	23.40 ± 3.01	9
				NCO	91	38/55 NA	55.94 ± 15.15	23.85 ± 3.15	
Lee H al. 2023	Retrospective PSM	2009–2016	Korea	CO	107	70/37	65.0(30.0–85.0)	22.7 (16.4–32.0)	9
				NCO	107	70/37	62.0(30.0–92.0)	23.5 (15.3–31.4)	
Lee S al. 2022	Retrospective PSM	2014–2018	Korea	CO	174	122/52	59.9 ± 12.7	23.1 ± 3.7	9
				NCO	248	177/71	61.6 ± 13.3	23.8 ± 3.1	
Seo WJ al. 2021	Retrospective PSM	2003–2015	Korea	CO	225	131/94	59 (49–70)	23.5 (21.1–25.6)	9
				NCO	225	137/88	56 (49–67)	22.9 (21.0–24.9)	
Murakami H al. 2021	Prospective RCT	2011–2018	Japan	CO	122	89/33	71 (30–90)	22.4 (14.8–31.8)	-
				NCO	125	89/36	74 (45–89)	22.2 (14.5–32.1)	
Sakimura Y al. 2020	Retrospective PSM	2008–2017	Japan	CO	70	46/24	65.0 (37–90)	22.2 (15.8–30.3)	8
				NCO	70	48/22	66.5 (42–94)	22.4 (16.4–32.6)	
Ri M al. 2020	Retrospective PSM	2006–2012	Japan	CO	263	176/87	66.7 ± 11.0	22.4 ± 3.6	9
				NCO	263	181/82	65.7 ± 12.9	22.5 ± 3.4	
Young S al. 2020	Retrospective	2008–2016	USA	CO	90	62/28	69.5 (62–77)	27.4 ± 6.1	8
				NCO	381	217/164	68 (58–76)	26.2 ± 5.3	
Kim DJ al. 2014	Retrospective PSM	2004–2011	Korea	CO	80	56/24	60.9 ± 11.2	NA	7
				NCO	66	50/16	62.2 ± 11.0	NA	
Hasegawa S al. 2013	Retrospective PSM	2000–2009	Japan	CO	98	72/26	69.0 (40–91)	NA	8
				NCO	98	72/26	68.7 (45–91)	NA	
Kim MC al. 2011	Retrospective	2005–2009	Korea	CO	20	17/3	58.2 ± 9.5	23.3 ± 2.3	8
				NCO	17	11/6	58.6 ± 10.1	23.6 ± 2.7	
Ha TK al. 2008	Retrospective	2004–2006	Korea	CO	992	681/311	57.0 ± 11.3	NA	7
				NCO	124	87/37	56.3 ± 11.3	NA	

*BMI *Body Mass Index, *CO *complete omentectomy, *NCO *non-complete omentectomy, *PSM *propensity score matching, *RCT *randomized controlled trial, *NA *not available, Quality Score, According to Newcastle–Ottawa Scale

**Table 2:** General population characteristics

Authors/Year	Group	Surgical approach		Surgical procedure		Pathological T stage					Pathological *N* stage				TNM stage				Lymphadenectomy			
		Open	MIS	Others	TG	T0	T1	T2	T3	T4	N0	N1	N2	N3	I	II	III	IV	D1	D1+	D2	D2+
Song M al. 2023	CO	0	50	50	0	0	0	0	22	28	18		32		NA	NA	NA	NA	0	0	50	0
	NCO	0	58	58	0	0	0	0	25	23	20		38		NA	NA	NA	NA	0	0	58	0
Jeong SA al. 2023	CO	NA	NA	NA	NA	0	0	0	0	91	NA	NA	NA	NA	NA	NA	NA	NA	NA	NA	NA	NA
	NCO	NA	NA	NA	NA	0	0	0	0	91	NA	NA	NA	NA	NA	NA	NA	NA	NA	NA	NA	NA
Lee et al. 2023	CO	0	107	107	0	0	0	0	61	46	56		51		NA	NA	NA	NA	0	1	106	0
	NCO	0	107	107	0	0	0	0	61	46	56		51		NA	NA	NA	NA	0	14	93	0
Lee et al. 2022	CO	0	174	101	73	0	0	22	78	74	42	19	39	74	10	43	121	0	NA	NA	NA	NA
	NCO	0	248	157	91	0	0	45	119	84	65	35	62	86	21	77	150	0	NA	NA	NA	NA
Seo et al. 2021	CO	69	156	167	58	0	0	0	100	125	75	42	42	66	0	95	130	0	0	25	200	0
	NCO	60	165	169	56	0	0	0	111	114	73	47	42	63	0	99	126	0	0	22	203	0
Murakami et al. 2021	CO	122	0	73	49	0	20	21	42	39	44	29	25	24	26	48	41	7	1	9	110	2
	NCO	125	0	81	44	0	31	21	31	42	54	25	17	29	38	40	40	7	2	13	107	3
Sakimura et al. 2020	CO	41	29	45	25	1	5	12	32	20	22	14	14	20	NA	NA	NA	NA	9		61	
	NCO	25	45	44	26	0	6	16	27	21	29	14	9	18	NA	NA	NA	NA	14		56	
Ri et al. 2020	CO	263	0	156	107	0	47		216		148		115		28	101	129	5	11	146	102	4
	NCO	263	0	151	112	0	48		215		145		118		29	96	131	7	8	146	103	6
Young et al. 2020	CO	NA	NA	31	25	NA	NA	NA	41		47		53		NA	NA	NA	NA	NA	NA	NA	NA
	NCO	NA	NA	140	99	NA	NA	NA	184		176		205		NA	NA	NA	NA	NA	NA	NA	NA
Kim et al. 2014	CO	0	80	61	19	0	0	28	52	0	40	14	13	13	17	39	24	0	0	2	78	0
	NCO	0	66	24	12	0	0	37	29	0	34	8	16	8	23	26	17	0	0	5	61	0
Hasegawa et al. 2013	CO	98	0	52	46	0	0	30	34	34	39	25	18	16	16	40	42	0	0	12	84	2
	NCO	84	14	61	37	0	0	34	30	34	41	23	17	17	21	36	41	0	0	13	83	2
Kim et al. 2011	CO	20	0	20	0	0	20	0	0	0	19	1		0	NA	NA	NA	NA	13		7	
	NCO	17	0	17	0	0	16	1	0	0	15	2		0	NA	NA	NA	NA	15		2	
Ha et al. 2008	CO	NA	NA	917	75	early gastric cancer					NA	NA	NA	NA	977	15			NA	NA	NA	NA
	NCO	NA	NA	99	25	early gastric cancer					NA	NA	NA	NA	124	0			NA	NA	NA	NA

*CO *complete omentectomy, *NCO *non-complete omentectomy, *MIS *minimally invasive surgery, *TG *Total gastrectomy, *NA *not available

### Meta-analysis of outcomes

#### Surgery-related outcomes

**Fig. 3:**
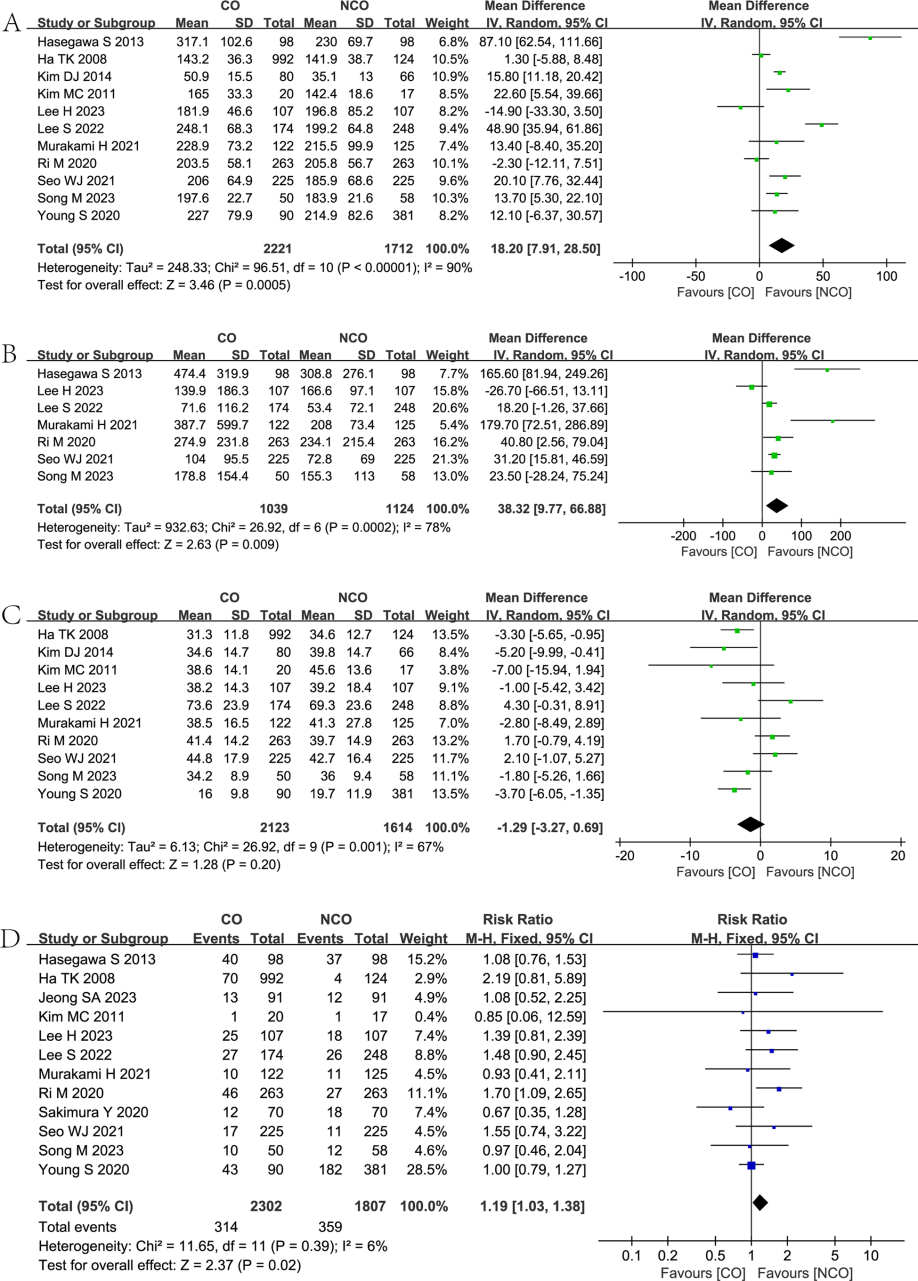
Forest plots showing the assessment of surgical-related outcomes: (**A**) operative time; (**B**) estimated blood loss; (**C**) the number of harvested lymph nodes; (**D**) postoperative complications. CO, complete omentectomy; NCO, non-complete omentectomy

Eleven studies [6–9, 11–15, 25, 27] reported the operative time in both groups, and the meta-analysis showed that the NCO group was associated with shorter operative time (MD = 18.20, 95% CI = 7.91–28.50, *P* = 0.0005) than the CO group. (Fig. 3A).

Seven studies [6–8, 11, 13, 25, 27] reported the estimated blood loss in both groups, and the meta-analysis showed that the NCO group was associated with lower estimated blood loss (MD = 38.32, 95% CI = 9.77–66.88, *P* = 0.009) than the CO group. (Fig. 3B).

Ten studies [6–9, 11, 12, 14, 15, 25, 27] reported the number of harvested lymph nodes in both groups, and the meta-analysis showed a non-statistically significant difference between the CO and NCO groups (MD = −1.29, 95% CI = −3.27–0.69, *P* = 0.20). (Fig. 3C).

Twelve studies [6–11, 13–15, 25–27] reported postoperative complications in both groups, and the meta-analysis showed that the NCO group was associated with fewer postoperative complications (RR = 1.19, 95% CI = 1.03–1.38, *P* = 0.02) than the CO group. (Fig. 3D).

#### Survival outcomes

**Fig. 4:**
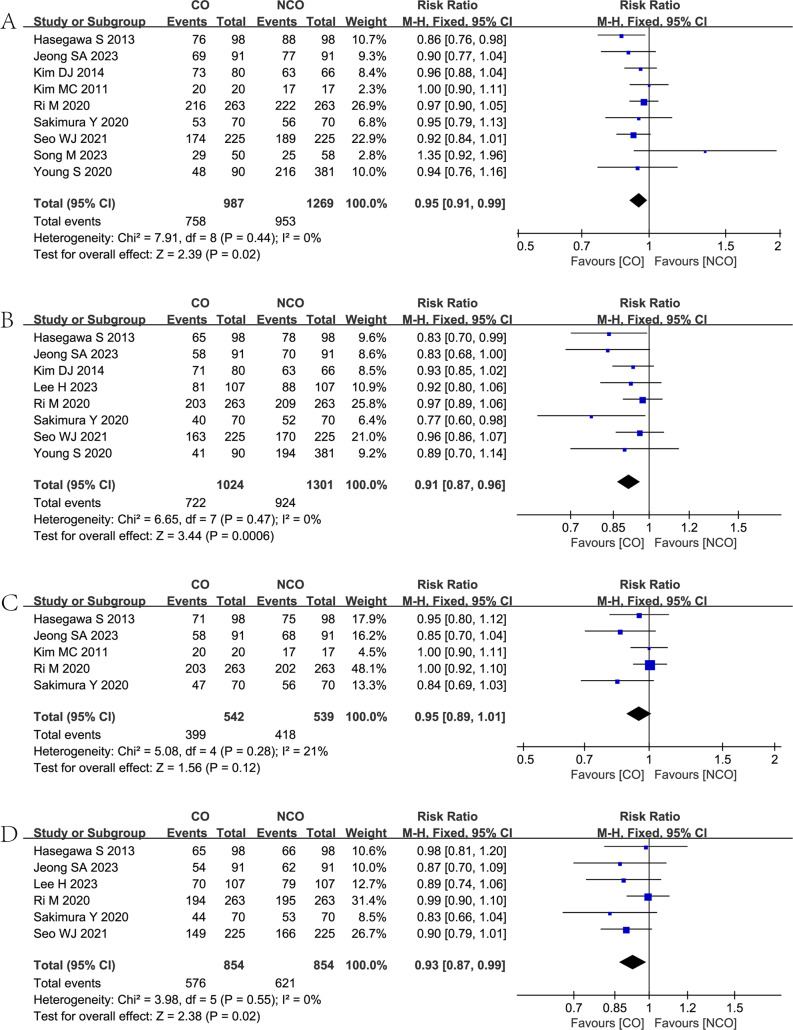
Forest plots showing the assessment of survival outcomes: (**A**) 3-year overall survival rate; (**B**) 5-year overall survival rate; (**C**) 3-year relapse-free survival rate; (**D**) 5-year relapse-free survival. CO, complete omentectomy; NCO, non-complete omentectomy

Nine studies [7, 9–14, 26, 27] reported the 3-year OS in both groups, and the meta-analysis showed that the NCO group was associated with a higher 3-year OS rate (RR = 0.95, 95% CI = 0.91–0.99, *P* = 0.02) than the CO group. (Fig. 4A).

Eight studies [7, 9–13, 25, 26] reported the 5-year OS in both groups, and the meta-analysis showed that the NCO group was associated with a higher 5-year OS rate (RR = 0.91, 95% CI = 0.87–0.96, *P* = 0.0006) than the CO group. (Fig. 4B).

Five studies [10, 11, 13, 14, 26] reported the 3-year RFS in both groups, and the meta-analysis showed that the difference between the two groups was not statistically significant. (RR = 0.95, 95% CI = 0.89–1.01, *P* = 0.12). (Fig. 4C).

Six studies [7, 10, 11, 13, 25, 26] reported the 5-year RFS in both groups, and the meta-analysis showed that the NCO group was associated with a higher 5-year RFS rate (RR = 0.93, 95% CI = 0.87–0.99, *P* = 0.02) than the CO group. (Fig. 4D).

#### Recurrence

**Fig. 5:**
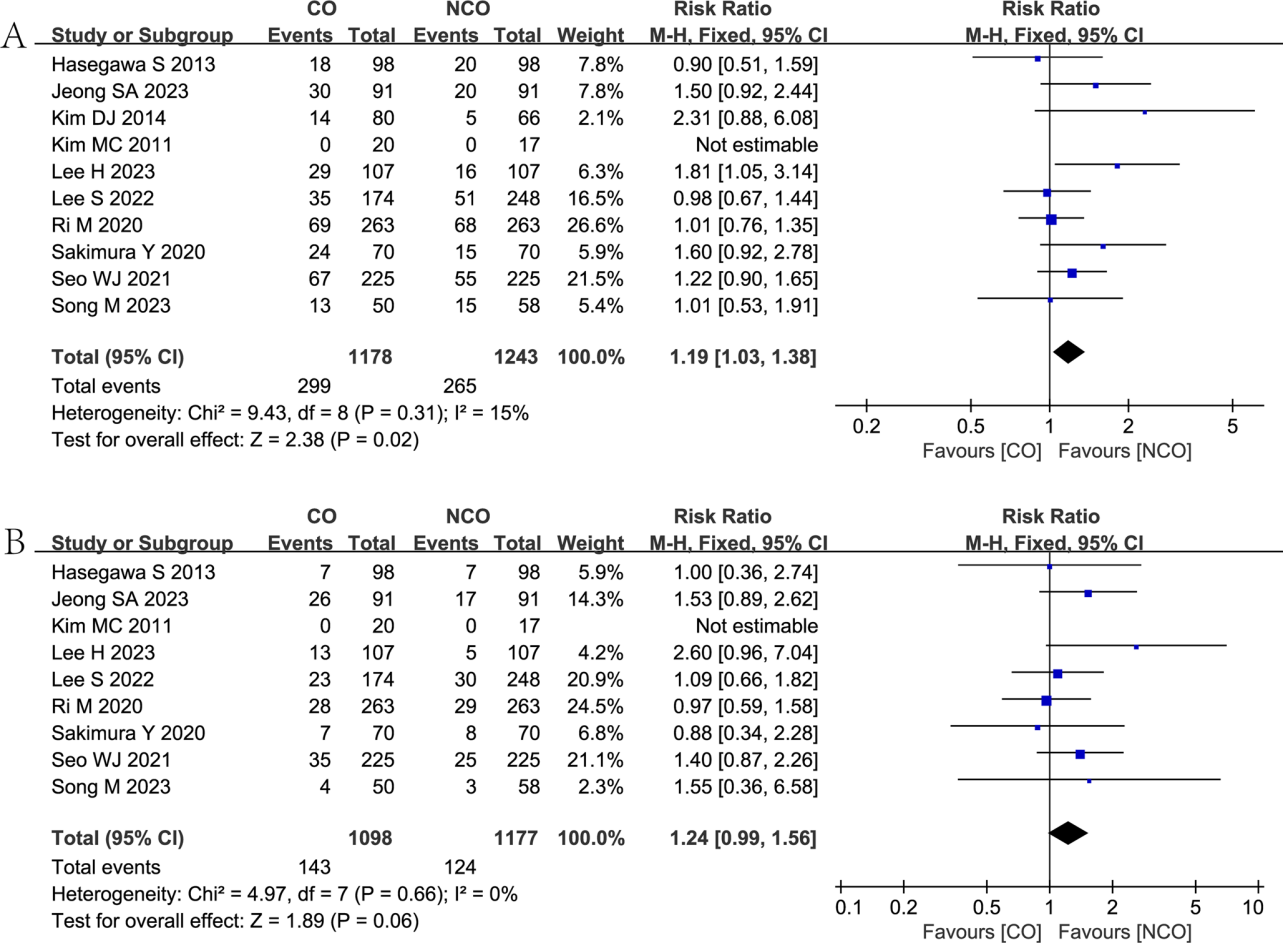
Forest plots showing the assessment of recurrence: (**A**) overall recurrence rate; (**B**) peritoneal recurrence rate. CO, complete omentectomy; NCO, non-complete omentectomy

Ten studies [6, 7, 10–14, 25–27] reported the overall recurrence rate in both groups, and the meta-analysis showed that the NCO group was associated with a lower overall recurrence rate (RR = 1.19, 95% CI = 1.03–1.38, *P* = 0.02) than the CO group. (Fig. 5A).

Nine studies [6, 7, 10, 11, 13, 14, 25–27] reported the peritoneal recurrence rate in both groups, and the meta-analysis showed no statistically significant difference in peritoneal recurrence rate (RR = 1.24, 95% CI = 0.99–1.56, *P* = 0.06) for the two groups. (Fig. 5B).

#### Subgroup analyses

Subgroup analyses were performed according to study design, and meta-analysis of PSM and RCT studies [6–8, 10–13, 25, 26] confirmed that the NCO group was associated with shorter operative times, fewer postoperative complications, lower rates of overall recurrence, higher 3-year and 5-year OS rates, and higher 5-year RFS rate. (Additional materials, Figures S1–10). Additionally, a subgroup analysis was conducted based on the quality of the research. A meta-analysis of retrospective studies with NOS scores of 9 and RCT studies confirmed that the NCO group was associated with lower estimated blood loss, fewer postoperative complications, lower rates of peritoneal recurrence, higher 5-year OS rates, and higher 5-year RFS rate. (Additional materials, Figures S11–20).

#### Publication bias

**Fig. 6:**
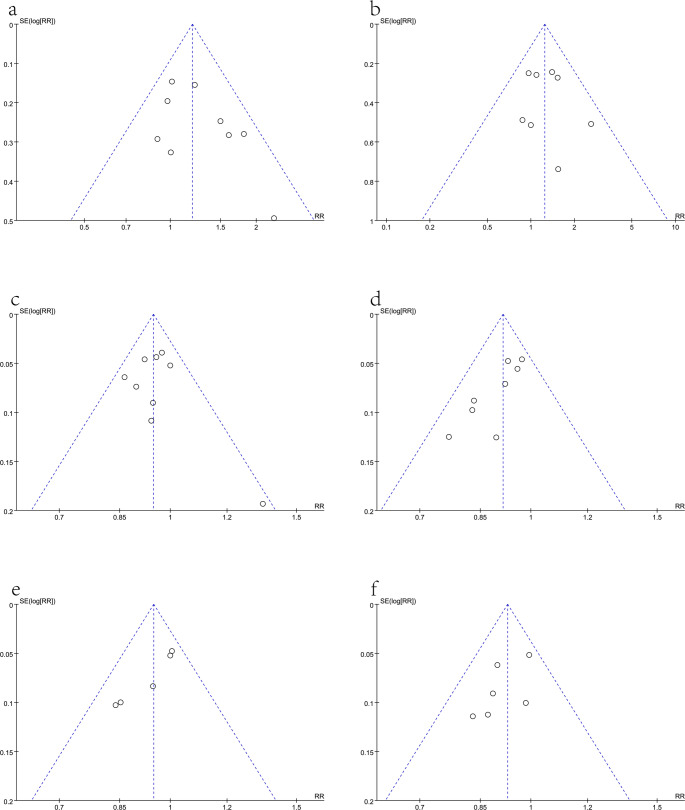
Funnel plots of publication bias: (**a**) Overall recurrence rate; (**b**) peritoneal recurrence rate; (**c**) 3-year OS rate; (**d**) 5-year OS rate; (**e**) 3-year RFS rate; (**f**) 5-year RFS rate Additional file 1.pdf. Title of data: Additional materials. Subgroup analysis by study type, including Figures S1–8

Our meta-analysis used funnel plots to assess publication bias (Fig. 6). Despite the symmetrical appearance of the funnel plot, the risk of publication bias was considered to be moderate due to the small number of RCTs included in the paper.

## Discussion

Surgery or endoscopic resection of GC is a necessary cornerstone of treatment [33]. However, after standard surgery, cancer still has a recurrence rate of 39–61% [34, 35]. The peritoneal recurrence rate accounts for 35% of the total recurrence rate [35]. Simultaneously, the peritoneum is one of the most common metastatic sites for GC, occurring in 15–30% of patients at diagnosis [36]. And it is estimated that this metastasis may occur in 55–60% of patients [37].

Among metastases to the peritoneum, metastasis to the greater omentum is a common site of GC metastasis, and this condition often signals a poor prognosis for the patient [38]. In anatomical terms, the greater omentum, adjacent to the stomach and colon, is a two-layered peritoneum that descends from the greater curvature of the stomach, covers the small intestine, and then folds up to fuse with the peritoneum on the anterior surface of the transverse colon, making the greater omentum susceptible to metastasis of cancerous cells implanted by gravity [39, 40]. Under microscopic observation, the greater omentum is composed of two different tissue types: a porous translucent membranous area and a vessel-rich area of adipose tissue, which stimulates the lymphatic tissue to accumulate around the blood vessels in an area known as the"milky spot “ [41]. The milky spot is composed of macrophages (70%), B cells (10%), T cells (10%), and mast cells [42]. Because it consists primarily of the above immune cells, it can function as a distinct secondary lymphoid tissue in peritoneal immunity [43]. With the exception of milk spots, the greater omentum is composed of mesothelial cells and vascular connective tissue. Mesothelial cells are sentinel cells that can sense and respond to signals within the microenvironment, and they play an important role in regulating inflammatory, immune, and tissue repair responses, and may even prevent the peritoneal spread of tumors [44]. The greater omentum has a certain anti-tumor effect due to the presence of milky spots and mesothelial cells. However, this structure is also susceptible to metastasis, and the milk spots and adipocytes can, under certain circumstances, be converted into a source of energy for tumor cells, as well as provide a suitable microenvironment for tumor cells [45]. Therefore, it is still controversial whether the omental tissue is completely resected in radical gastrectomy.

The meta-analysis in this paper was conducted to update the data related to the impact of NCO on patient outcomes compared to CO in radical gastrectomy. A total of thirteen studies including twelve retrospective studies and one RCT were included in this paper. Eight of the 12 retrospective studies progressed to PSM. Studies have shown that NCO is significantly associated with shorter operative time, less intraoperative bleeding, fewer postoperative complications, higher rates of 3-year and 5-year overall survival and 5-year recurrence-free survival, and a lower rate of postoperative recurrence. Subsequently, subgroup analysis was conducted based on the research design, and the results were identified as consistent with the previous findings. However, the results of the study indicated a notable statistical heterogeneity in relation to operative time, estimated blood loss, and the number of harvested lymph nodes. Three potential explanations for this heterogeneity can be postulated. Firstly, the duration of the study is 21 years, and the surgical approaches employed in each study may vary, resulting in discrepancies in the selection of anastomosis techniques and surgical equipment by the researchers. Secondly, the studies included GC patients with varying clinical and pathological stages. Thirdly, the inclusion of predominantly retrospective studies may result in heterogeneity due to measurement errors.

The result of this paper indicates that the NCO cohort demonstrated higher OS and 5-year RFS rates, which may potentially be attributed to the following factors: CO increases the difficulty of the operation due to the need to handle more omentum, particularly in laparoscopic surgery. The resection involves more blood vessels and tissue structures, which, under the same conditions, prolongs the surgical time and increases the possibility of intraoperative bleeding. Previous research indicates that increased intraoperative blood loss may negatively impact the prognosis of GC patients [46]. However, the absence of the omentum could reduce the body’s ability to limit infection and prevent disease [44], potentially leading to more complications and a poorer prognosis. In terms of recurrence rate, it is noteworthy that the NCO group is significantly correlated with a lower overall recurrence rate. However, no significant difference was observed in the results of peritoneal recurrence between the two groups. Firstly, the surgical procedure in the CO group is associated with greater blood loss and a longer surgical duration, which might increase the risk of damaging the integrity of lymphatic and blood vessels in the surgical area. Furthermore, it is important to consider the role of the omentum in regulating inflammatory, immune, and tissue repair responses when removing it during gastric cancer surgery. The combined effect of two distinct types of injury factors may potentially exacerbate the possibility of recurrence in GC patients during the recovery period.

For patients with advanced gastric cancer, the optimal treatment plan should include not only surgical intervention but also a multimodal treatment to improve prognosis [47]. Although the treatment guidelines for adjuvant chemotherapy and neoadjuvant chemotherapy vary in different countries, adjuvant chemotherapy is recommended in Asian countries, while neoadjuvant chemotherapy and adjuvant chemotherapy are recommended in Europe and America [48, 49]. With the confirmation of the safety and efficacy of neoadjuvant chemotherapy, the application of adjuvant therapy may be of greater importance than the selection of omentectomy. Among the studies included in the paper, seven studies [6, 7, 10, 11, 13, 26, 27] mentioned the use of chemotherapy, but did not provide specific prognostic data. Consequently, it is imperative to obtain more comprehensive data regarding the entirety of the cancer treatment process in order to reliably reach conclusions.

Although thirteen relevant studies were included in our paper, the impact of preoperative pathologic staging and tumor location of GC on surgical, survival, and recurrence outcomes was not considered. Perioperative chemotherapy has been the standard of care for resectable localized GC, while adjuvant chemotherapy is recommended for patients with stage II or III disease who have improved survival [3]. The impact of these treatments on patients was not explored by us. Furthermore, the majority of studies originate from Asian countries, which introduces certain limitations. Therefore, we cannot completely exclude whether the above factors might have influenced the differences between the CO and NCO groups.

## Conclusions

In conclusion, our meta-analysis indicates that compared to NCO, CO has no benefit on surgery-related, survival, or recurrence outcomes. Although there are limitations to the studies discussed in this paper, based on the available evidence, NCO may be a more feasible procedure in radical gastrectomy. As most of the studies included in our meta-analysis were retrospective, more high-quality and well-designed RCTs are needed to further substantiate our results.

## Electronic supplementary material

Below is the link to the electronic supplementary material.

Supplementary Material 1

## Data Availability

No datasets were generated or analysed during the current study.

## Abbreviations

NCO: Non-complete omentectomy
CO: Complete omentectomy
OS: Overall survival
RFS: Relapse-free survival
GC: Gastric cancer
PRISMA: Preferred Reporting Items for Systematic Reviews and Meta-Analyses
RCT: Randomized controlled trial
RRs: Risk ratios
CIs: Confidence intervals
MDs: Mean differences
SD: Standard deviation
MH: Mantel–Haenszel
PSM: Propensity score-matching
