# Supplementary material for: The efficacy of non-complete omentectomy in the radical gastrectomy for gastric cancer: a meta-analysis and systematic review
Source: Langenbecks Arch Surg. 2025 Jun 3;410(1):173. doi: 10.1007/s00423-025-03760-2 (PMC12133918; doi:10.1007/s00423-025-03760-2)
Supplement: Supplementary file 1 — Supplementary Material 1 [file 423_2025_3760_MOESM1_ESM.pdf]

Additional materials

Study or Subgroup	CO Mean	CO SD	CO Total	NCO Mean	NCO SD	NCO Total	Weight	Mean Difference IV, Random, 95% CI
Hasegawa S 2013	317.1	102.6	98	230	69.7	98	12.0%	87.10 [62.54, 111.66]
Kim DJ 2014	50.9	15.5	80	35.1	13	66	16.2%	15.80 [11.18, 20.42]
Lee H 2023	181.9	46.6	107	196.8	85.2	107	13.6%	-14.90 [-33.30, 3.50]
Lee S 2022	248.1	68.3	174	199.2	64.8	248	14.9%	48.90 [35.94, 61.86]
Murakami H 2021	228.9	73.2	122	215.5	99.9	125	12.8%	13.40 [-8.40, 35.20]
Ri M 2020	203.5	58.1	263	205.8	56.7	263	15.5%	-2.30 [-12.11, 7.51]
Seo WJ 2021	206	64.9	225	185.9	68.6	225	15.0%	20.10 [7.76, 32.44]
**Total (95% CI)**			**1069**			**1132**	**100.0%**	**22.67 [6.11, 39.23]**

Heterogeneity: Tau² = 435.84; Chi² = 81.53, df = 6 (P < 0.00001); I² = 93%
Test for overall effect: Z = 2.68 (P = 0.007)

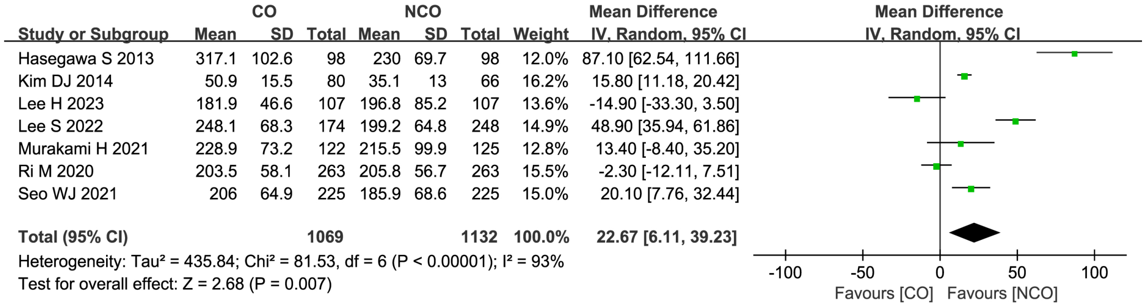

Figure S 1. Forest plot of operative time in subgroup analysis1. [6-8, 11, 12, 14, 25]

Study or Subgroup	CO Mean	CO SD	CO Total	NCO Mean	NCO SD	NCO Total	Weight	Mean Difference IV, Random, 95% CI
Hasegawa S 2013	474.4	319.9	98	308.8	276.1	98	9.3%	165.60 [81.94, 249.26]
Lee H 2023	139.9	186.3	107	166.6	97.1	107	18.3%	-26.70 [-66.51, 13.11]
Lee S 2022	71.6	116.2	174	53.4	72.1	248	23.2%	18.20 [-1.26, 37.66]
Murakami H 2021	387.7	599.7	122	208	73.4	125	6.6%	179.70 [72.51, 286.89]
Ri M 2020	274.9	231.8	263	234.1	215.4	263	18.7%	40.80 [2.56, 79.04]
Seo WJ 2021	104	95.5	225	72.8	69	225	23.9%	31.20 [15.81, 46.59]
**Total (95% CI)**			**989**			**1066**	**100.0%**	**41.84 [9.65, 74.03]**

Heterogeneity: Tau² = 1065.26; Chi² = 26.90, df = 5 (P < 0.0001); I² = 81%
Test for overall effect: Z = 2.55 (P = 0.01)

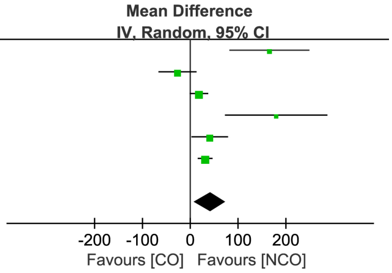

Figure S 2. Forest plot of estimated blood loss in subgroup analysis1. [6-8, 11, 13, 25]

Study or Subgroup	CO Mean	CO SD	CO Total	NCO Mean	NCO SD	NCO Total	Weight	Mean Difference IV, Random, 95% CI
Kim DJ 2014	34.6	14.7	80	39.8	14.7	66	14.2%	-5.20 [-9.99, -0.41]
Lee H 2023	38.2	14.3	107	39.2	18.4	107	15.5%	-1.00 [-5.42, 3.42]
Lee S 2022	73.6	23.9	174	69.3	23.6	248	14.8%	4.30 [-0.31, 8.91]
Murakami H 2021	38.5	16.5	122	41.3	27.8	125	11.7%	-2.80 [-8.49, 2.89]
Ri M 2020	41.4	14.2	263	39.7	14.9	263	23.4%	1.70 [-0.79, 4.19]
Seo WJ 2021	44.8	17.9	225	42.7	16.4	225	20.4%	2.10 [-1.07, 5.27]
**Total (95% CI)**			**971**			**1034**	**100.0%**	**0.24 [-2.22, 2.71]**

Heterogeneity: Tau² = 5.13; Chi² = 11.56, df = 5 (P = 0.04); I² = 57%
Test for overall effect: Z = 0.19 (P = 0.85)

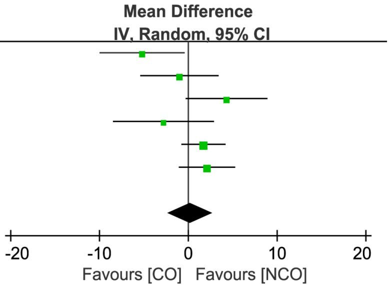

Figure S 3. Forest plot of the number of harvested lymph nodes in subgroup analysis1. [6-8, 11, 12, 25]

Study or Subgroup	CO Events	CO Total	NCO Events	NCO Total	Weight	Risk Ratio M-H, Fixed, 95% CI
Hasegawa S 2013	40	98	37	98	23.8%	1.08 [0.76, 1.53]
Jeong SA 2023	13	91	12	91	7.7%	1.08 [0.52, 2.25]
Lee H 2023	25	107	18	107	11.6%	1.39 [0.81, 2.39]
Lee S 2022	27	174	26	248	13.8%	1.48 [0.90, 2.45]
Murakami H 2021	10	122	11	125	7.0%	0.93 [0.41, 2.11]
Ri M 2020	46	263	27	263	17.4%	1.70 [1.09, 2.65]
Sakimura Y 2020	12	70	18	70	11.6%	0.67 [0.35, 1.28]
Seo WJ 2021	17	225	11	225	7.1%	1.55 [0.74, 3.22]
**Total (95% CI)**		**1150**		**1227**	**100.0%**	**1.25 [1.04, 1.52]**
Total events	190		160			

Heterogeneity: Chi² = 7.68, df = 7 (P = 0.36); I² = 9%
Test for overall effect: Z = 2.36 (P = 0.02)

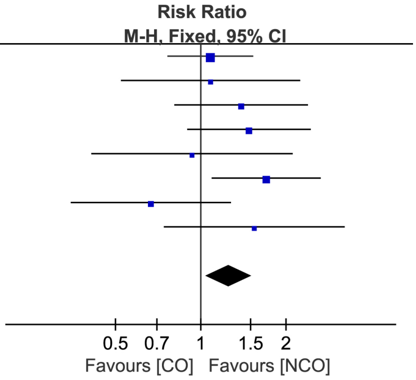

Figure S 4. Forest plot of postoperative complications in subgroup analysis1. [6-8, 10, 11, 13, 25, 26]

Study or Subgroup	CO Events	CO Total	NCO Events	NCO Total	Weight	Risk Ratio M-H, Fixed, 95% CI
Hasegawa S 2013	76	98	88	98	12.6%	0.86 [0.76, 0.98]
Jeong SA 2023	69	91	77	91	11.0%	0.90 [0.77, 1.04]
Kim DJ 2014	73	80	63	66	9.8%	0.96 [0.88, 1.04]
Ri M 2020	216	263	222	263	31.7%	0.97 [0.90, 1.05]
Sakimura Y 2020	53	70	56	70	8.0%	0.95 [0.79, 1.13]
Seo WJ 2021	174	225	189	225	27.0%	0.92 [0.84, 1.01]
**Total (95% CI)**		**827**		**813**	**100.0%**	**0.93 [0.89, 0.98]**
Total events	661		695			

Heterogeneity: Chi² = 3.31, df = 5 (P = 0.65); I² = 0%
Test for overall effect: Z = 3.08 (P = 0.002)

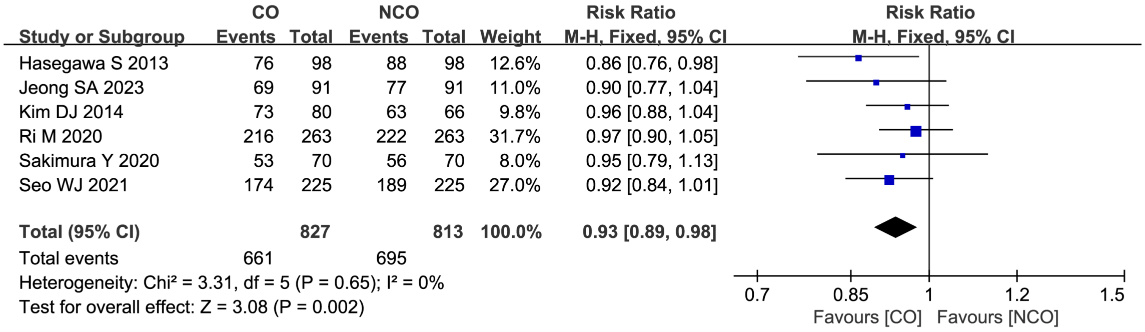

Figure S 5. Forest plot of the 3-year OS in subgroup analysis1. [7, 10-13, 26]

Study or Subgroup	CO Events	CO Total	NCO Events	NCO Total	Weight	Risk Ratio M-H, Fixed, 95% CI
Hasegawa S 2013	65	98	78	98	10.6%	0.83 [0.70, 0.99]
Jeong SA 2023	58	91	70	91	9.5%	0.83 [0.68, 1.00]
Kim DJ 2014	71	80	63	66	9.4%	0.93 [0.85, 1.02]
Lee H 2023	81	107	88	107	12.0%	0.92 [0.80, 1.06]
Ri M 2020	203	263	209	263	28.4%	0.97 [0.89, 1.06]
Sakimura Y 2020	40	70	52	70	7.1%	0.77 [0.60, 0.98]
Seo WJ 2021	163	225	170	225	23.1%	0.96 [0.86, 1.07]
**Total (95% CI)**		**934**		**920**	**100.0%**	**0.92 [0.87, 0.96]**
Total events	681		730			

Heterogeneity: Chi² = 6.55, df = 6 (P = 0.36); I² = 8%
Test for overall effect: Z = 3.39 (P = 0.0007)

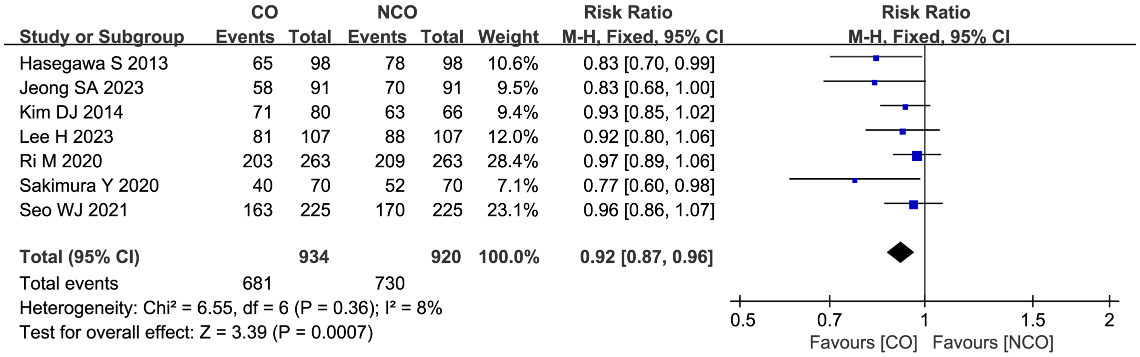

Figure S 6. Forest plot of the 5-year OS in subgroup analysis1. [7, 10-13, 25, 26]

Study or Subgroup	CO Events	CO Total	NCO Events	NCO Total	Weight	Risk Ratio M-H, Fixed, 95% CI
Hasegawa S 2013	71	98	75	91	19.3%	0.88 [0.75, 1.03]
Jeong SA 2023	58	91	68	91	16.8%	0.85 [0.70, 1.04]
Ri M 2020	203	263	202	263	50.0%	1.00 [0.92, 1.10]
Sakimura Y 2020	47	70	56	70	13.9%	0.84 [0.69, 1.03]
**Total (95% CI)**		**522**		**515**	**100.0%**	**0.93 [0.87, 1.00]**
Total events	379		401			

Heterogeneity: Chi² = 4.88, df = 3 (P = 0.18); I² = 38%
Test for overall effect: Z = 1.97 (P = 0.05)

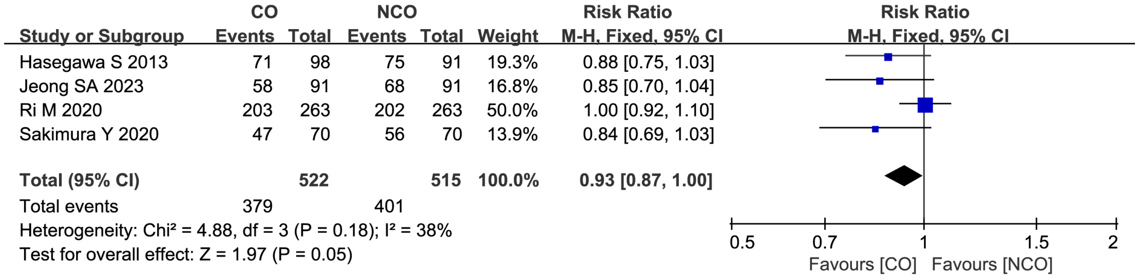

Figure S 7. Forest plot of the 3-year RFS in subgroup analysis1. [10, 11, 13, 26]

Study or Subgroup	CO Events	CO Total	NCO Events	NCO Total	Weight	Risk Ratio M-H, Fixed, 95% CI
Hasegawa S 2013	65	98	66	98	10.6%	0.98 [0.81, 1.20]
Jeong SA 2023	54	91	62	91	10.0%	0.87 [0.70, 1.09]
Lee H 2023	70	107	79	107	12.7%	0.89 [0.74, 1.06]
Ri M 2020	194	263	195	263	31.4%	0.99 [0.90, 1.10]
Sakimura Y 2020	44	70	53	70	8.5%	0.83 [0.66, 1.04]
Seo WJ 2021	149	225	166	225	26.7%	0.90 [0.79, 1.01]
**Total (95% CI)**		**854**		**854**	**100.0%**	**0.93 [0.87, 0.99]**
Total events	576		621			

Heterogeneity: Chi² = 3.98, df = 5 (P = 0.55); I² = 0%
Test for overall effect: Z = 2.38 (P = 0.02)

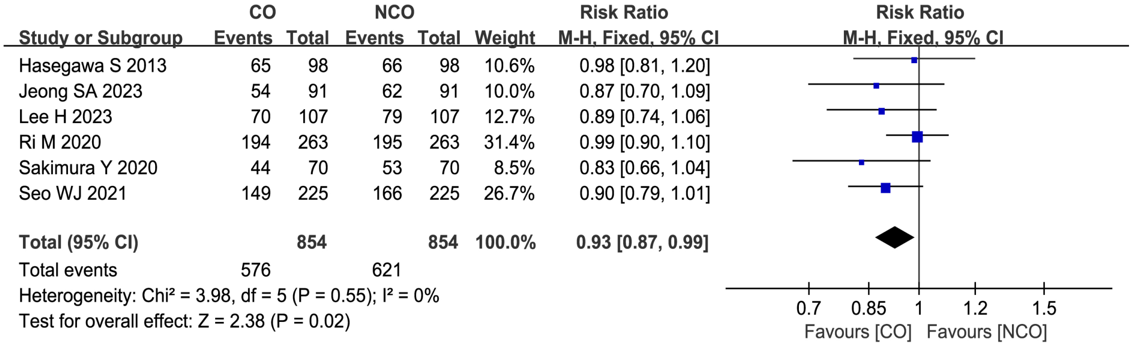

Figure S 8. Forest plot of the 5-year RFS in subgroup analysis1. [7, 10, 11, 13, 25, 26]

Study or Subgroup	CO Events	CO Total	NCO Events	NCO Total	Weight	Risk Ratio M-H, Fixed, 95% CI
Hasegawa S 2013	18	98	20	98	8.3%	0.90 [0.51, 1.59]
Jeong SA 2023	30	91	20	91	8.3%	1.50 [0.92, 2.44]
Kim DJ 2014	14	80	5	66	2.3%	2.31 [0.88, 6.08]
Lee H 2023	29	107	16	107	6.6%	1.81 [1.05, 3.14]
Lee S 2022	35	174	51	248	17.4%	0.98 [0.67, 1.44]
Ri M 2020	69	263	68	263	28.2%	1.01 [0.76, 1.35]
Sakimura Y 2020	24	70	15	70	6.2%	1.60 [0.92, 2.78]
Seo WJ 2021	67	225	55	225	22.8%	1.22 [0.90, 1.65]
**Total (95% CI)**		**1108**		**1168**	**100.0%**	**1.20 [1.04, 1.40]**
Total events	286		250			

Heterogeneity: Chi² = 9.16, df = 7 (P = 0.24); I² = 24%
Test for overall effect: Z = 2.44 (P = 0.01)

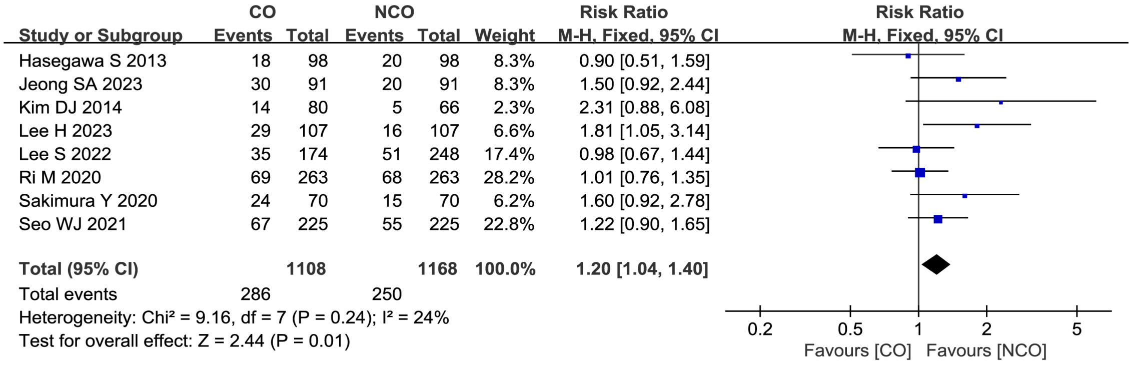

Figure S 9. Forest plot of the overall recurrence rate in subgroup analysis1. [6, 7, 10-13, 25, 26]

Study or Subgroup	CO Events	CO Total	NCO Events	NCO Total	Weight	Risk Ratio M-H, Fixed, 95% CI
Hasegawa S 2013	7	98	7	98	6.0%	1.00 [0.36, 2.74]
Jeong SA 2023	26	91	17	91	14.7%	1.53 [0.89, 2.62]
Lee H 2023	13	107	5	107	4.3%	2.60 [0.96, 7.04]
Lee S 2022	23	174	30	248	21.4%	1.09 [0.66, 1.82]
Ri M 2020	28	263	29	263	25.1%	0.97 [0.59, 1.58]
Sakimura Y 2020	7	70	8	70	6.9%	0.88 [0.34, 2.28]
Seo WJ 2021	35	225	25	225	21.6%	1.40 [0.87, 2.26]
**Total (95% CI)**		**1028**		**1102**	**100.0%**	**1.24 [0.98, 1.55]**
Total events	139		121			

Heterogeneity: Chi² = 4.87, df = 6 (P = 0.56); I² = 0%
Test for overall effect: Z = 1.82 (P = 0.07)

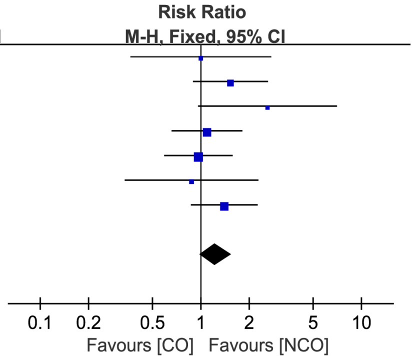

Figure S 10. Forest plot of the peritoneal recurrence rate in subgroup analysis1. [6, 7, 10, 11, 13, 25, 26]

Study or Subgroup	CO Mean	CO SD	CO Total	NCO Mean	NCO SD	NCO Total	Weight	Mean Difference IV, Random, 95% CI
Lee H 2023	181.9	46.6	107	196.8	85.2	107	15.3%	-14.90 [-33.30, 3.50]
Lee S 2022	248.1	68.3	174	199.2	64.8	248	17.1%	48.90 [35.94, 61.86]
Murakami H 2021	228.9	73.2	122	215.5	99.9	125	14.2%	13.40 [-8.40, 35.20]
Ri M 2020	203.5	58.1	263	205.8	56.7	263	17.9%	-2.30 [-12.11, 7.51]
Seo WJ 2021	206	64.9	225	185.9	68.6	225	17.2%	20.10 [7.76, 32.44]
Song M 2023	197.6	22.7	50	183.9	21.6	58	18.2%	13.70 [5.30, 22.10]
**Total (95% CI)**			**941**			**1026**	**100.0%**	**13.52 [-2.55, 29.58]**

Heterogeneity: Tau² = 349.78; Chi² = 48.86, df = 5 (P < 0.00001); I² = 90%
Test for overall effect: Z = 1.65 (P = 0.10)

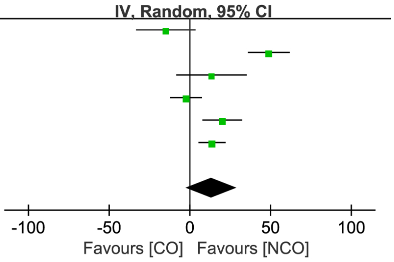

Figure S 11. Forest plot of operative time in subgroup analysis2. [6-8, 11, 25, 27]

Study or Subgroup	CO Mean	CO SD	CO Total	NCO Mean	NCO SD	NCO Total	Weight	Mean Difference IV, Random, 95% CI
Lee H 2023	139.9	186.3	107	166.6	97.1	107	16.2%	-26.70 [-66.51, 13.11]
Lee S 2022	71.6	116.2	174	53.4	72.1	248	24.4%	18.20 [-1.26, 37.66]
Murakami H 2021	387.7	599.7	122	208	73.4	125	4.3%	179.70 [72.51, 286.89]
Ri M 2020	274.9	231.8	263	234.1	215.4	263	16.7%	40.80 [2.56, 79.04]
Seo WJ 2021	104	95.5	225	72.8	69	225	26.0%	31.20 [15.81, 46.59]
Song M 2023	178.8	154.4	50	155.3	113	58	12.4%	23.50 [-28.24, 75.24]
**Total (95% CI)**			**941**			**1026**	**100.0%**	**25.68 [1.64, 49.72]**

Heterogeneity: Tau² = 517.57; Chi² = 16.23, df = 5 (P = 0.006); I² = 69%
Test for overall effect: Z = 2.09 (P = 0.04)

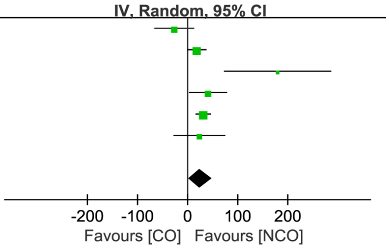

Figure S 12. Forest plot of estimated blood loss in subgroup analysis2. [6-8, 11, 25, 27]

Study or Subgroup	CO Mean	CO SD	CO Total	NCO Mean	NCO SD	NCO Total	Weight	Mean Difference IV, Random, 95% CI
Lee H 2023	38.2	14.3	107	39.2	18.4	107	13.3%	-1.00 [-5.42, 3.42]
Lee S 2022	73.6	23.9	174	69.3	23.6	248	12.5%	4.30 [-0.31, 8.91]
Murakami H 2021	38.5	16.5	122	41.3	27.8	125	9.0%	-2.80 [-8.49, 2.89]
Ri M 2020	41.4	14.2	263	39.7	14.9	263	26.3%	1.70 [-0.79, 4.19]
Seo WJ 2021	44.8	17.9	225	42.7	16.4	225	20.5%	2.10 [-1.07, 5.27]
Song M 2023	34.2	8.9	50	36	9.4	58	18.5%	-1.80 [-5.26, 1.66]
**Total (95% CI)**			**941**			**1026**	**100.0%**	**0.70 [-1.19, 2.58]**

Heterogeneity: Tau² = 1.90; Chi² = 7.72, df = 5 (P = 0.17); I² = 35%
Test for overall effect: Z = 0.72 (P = 0.47)

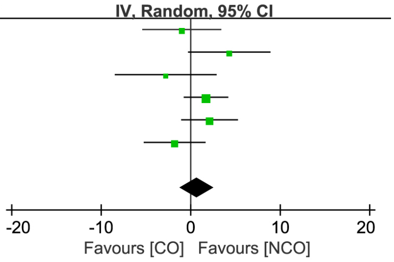

Figure S 13. Forest plot of the number of harvested lymph nodes in subgroup analysis2. [6-8, 11, 25, 27]

Study or Subgroup	CO Events	CO Total	NCO Events	NCO Total	Weight	Risk Ratio M-H, Fixed, 95% CI
Jeong SA 2023	13	91	12	91	10.8%	1.08 [0.52, 2.25]
Lee H 2023	25	107	18	107	16.2%	1.39 [0.81, 2.39]
Lee S 2022	27	174	26	248	19.2%	1.48 [0.90, 2.45]
Murakami H 2021	10	122	11	125	9.8%	0.93 [0.41, 2.11]
Ri M 2020	46	263	27	263	24.2%	1.70 [1.09, 2.65]
Seo WJ 2021	17	225	11	225	9.9%	1.55 [0.74, 3.22]
Song M 2023	10	50	12	58	10.0%	0.97 [0.46, 2.04]
**Total (95% CI)**		**1032**		**1117**	**100.0%**	**1.38 [1.10, 1.73]**
Total events	148		117			

Heterogeneity: Chi² = 3.21, df = 6 (P = 0.78); I² = 0%
Test for overall effect: Z = 2.78 (P = 0.005)

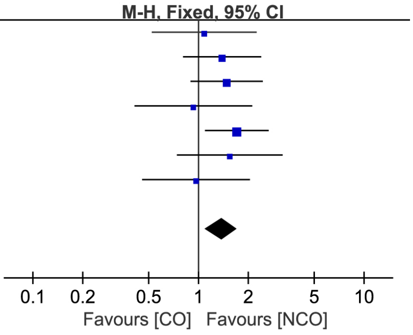

Figure S 14. Forest plot of postoperative complications in subgroup analysis2. [6-8, 11, 25-27]

Study or Subgroup	CO Events	CO Total	NCO Events	NCO Total	Weight	Risk Ratio M-H, Fixed, 95% CI
Jeong SA 2023	69	91	77	91	15.1%	0.90 [0.77, 1.04]
Ri M 2020	216	263	222	263	43.4%	0.97 [0.90, 1.05]
Seo WJ 2021	174	225	189	225	37.0%	0.92 [0.84, 1.01]
Song M 2023	29	50	25	58	4.5%	1.35 [0.92, 1.96]
**Total (95% CI)**		**629**		**637**	**100.0%**	**0.96 [0.91, 1.01]**
Total events	488		513			

Heterogeneity: Chi² = 4.83, df = 3 (P = 0.18); I² = 38%
Test for overall effect: Z = 1.48 (P = 0.14)

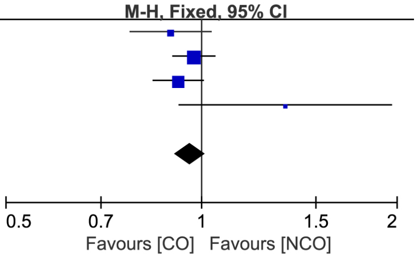

Figure S 15. Forest plot of the 3-year OS in subgroup analysis2. [7, 11, 26, 27]

Study or Subgroup	CO Events	CO Total	NCO Events	NCO Total	Weight	Risk Ratio M-H, Fixed, 95% CI
Jeong SA 2023	58	91	70	91	13.0%	0.83 [0.68, 1.00]
Lee H 2023	81	107	88	107	16.4%	0.92 [0.80, 1.06]
Ri M 2020	203	263	209	263	38.9%	0.97 [0.89, 1.06]
Seo WJ 2021	163	225	170	225	31.7%	0.96 [0.86, 1.07]
**Total (95% CI)**		**686**		**686**	**100.0%**	**0.94 [0.89, 1.00]**
Total events	505		537			

Heterogeneity: Chi² = 2.39, df = 3 (P = 0.50); I² = 0%
Test for overall effect: Z = 2.02 (P = 0.04)

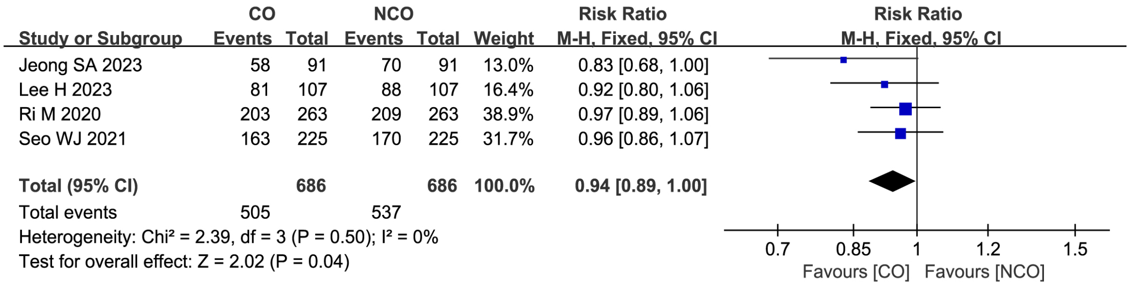

Figure S 16. Forest plot of the 5-year OS in subgroup analysis2. [7, 11, 25, 26]

Study or Subgroup	CO Events	CO Total	NCO Events	NCO Total	Weight	Risk Ratio M-H, Fixed, 95% CI
Jeong SA 2023	58	91	68	91	25.2%	0.85 [0.70, 1.04]
Ri M 2020	203	263	202	263	74.8%	1.00 [0.92, 1.10]
**Total (95% CI)**		**354**		**354**	**100.0%**	**0.97 [0.89, 1.05]**
Total events	261		270			

Heterogeneity: Chi² = 2.24, df = 1 (P = 0.13); I² = 55%
Test for overall effect: Z = 0.78 (P = 0.43)

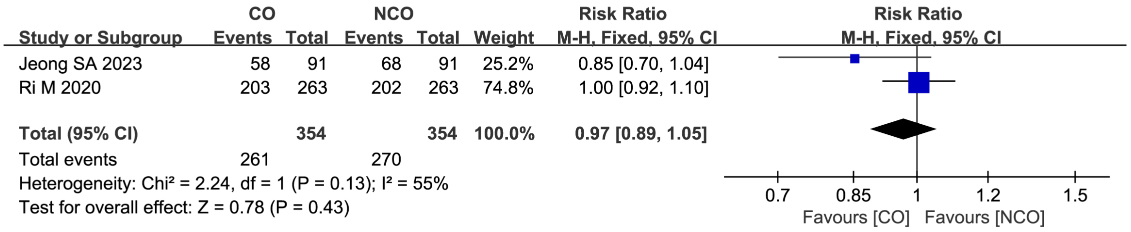

Figure S 17. Forest plot of the 3-year RFS in subgroup analysis2. [11, 26]

Study or Subgroup	CO Events	CO Total	NCO Events	NCO Total	Weight	Risk Ratio M-H, Fixed, 95% CI
Jeong SA 2023	54	91	62	91	12.4%	0.87 [0.70, 1.09]
Lee H 2023	70	107	79	107	15.7%	0.89 [0.74, 1.06]
Ri M 2020	194	263	195	263	38.8%	0.99 [0.90, 1.10]
Seo WJ 2021	149	225	166	225	33.1%	0.90 [0.79, 1.01]
**Total (95% CI)**		**686**		**686**	**100.0%**	**0.93 [0.87, 1.00]**
Total events	467		502			

Heterogeneity: Chi² = 2.64, df = 3 (P = 0.45); I² = 0%
Test for overall effect: Z = 2.07 (P = 0.04)

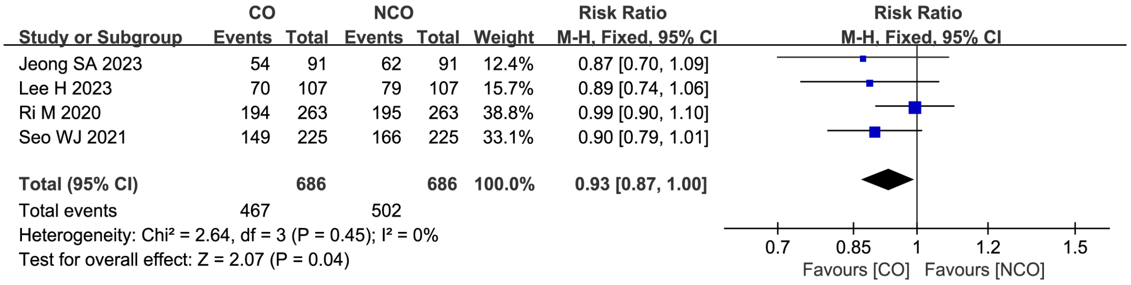

Figure S 18. Forest plot of the 5-year RFS in subgroup analysis2. [7, 11, 25, 26]

Study or Subgroup	CO Events	CO Total	NCO Events	NCO Total	Weight	Risk Ratio M-H, Fixed, 95% CI
Jeong SA 2023	30	91	20	91	9.3%	1.50 [0.92, 2.44]
Lee H 2023	29	107	16	107	7.4%	1.81 [1.05, 3.14]
Lee S 2022	35	174	51	248	19.6%	0.98 [0.67, 1.44]
Ri M 2020	69	263	68	263	31.6%	1.01 [0.76, 1.35]
Seo WJ 2021	67	225	55	225	25.6%	1.22 [0.90, 1.65]
Song M 2023	13	50	15	58	6.5%	1.01 [0.53, 1.91]
**Total (95% CI)**		**910**		**992**	**100.0%**	**1.16 [0.99, 1.36]**
Total events	243		225			

Heterogeneity: Chi² = 5.50, df = 5 (P = 0.36); I² = 9%
Test for overall effect: Z = 1.88 (P = 0.06)

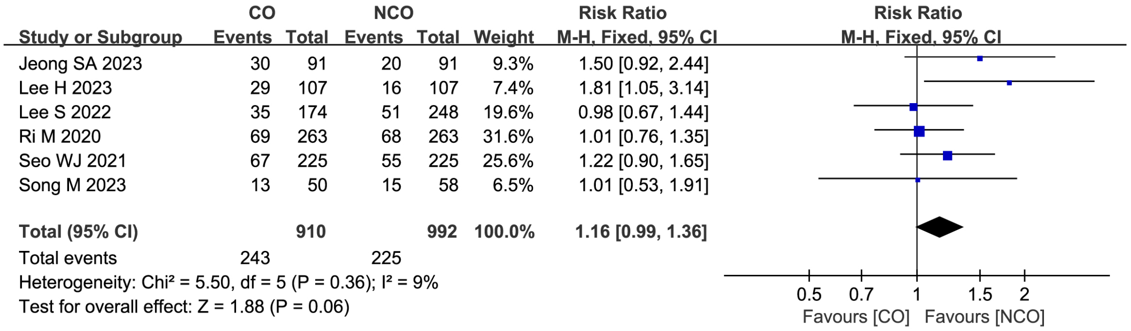

Figure S 19. Forest plot of the overall recurrence rate in subgroup analysis2. [6, 7, 11, 25-27]

Study or Subgroup	CO Events	CO Total	NCO Events	NCO Total	Weight	Risk Ratio M-H, Fixed, 95% CI
Jeong SA 2023	26	91	17	91	16.4%	1.53 [0.89, 2.62]
Lee H 2023	13	107	5	107	4.8%	2.60 [0.96, 7.04]
Lee S 2022	23	174	30	248	23.9%	1.09 [0.66, 1.82]
Ri M 2020	28	263	29	263	28.0%	0.97 [0.59, 1.58]
Seo WJ 2021	35	225	25	225	24.2%	1.40 [0.87, 2.26]
Song M 2023	4	50	3	58	2.7%	1.55 [0.36, 6.58]
**Total (95% CI)**		**910**		**992**	**100.0%**	**1.29 [1.01, 1.63]**
Total events	129		109			

Heterogeneity: Chi² = 4.21, df = 5 (P = 0.52); I² = 0%
Test for overall effect: Z = 2.08 (P = 0.04)

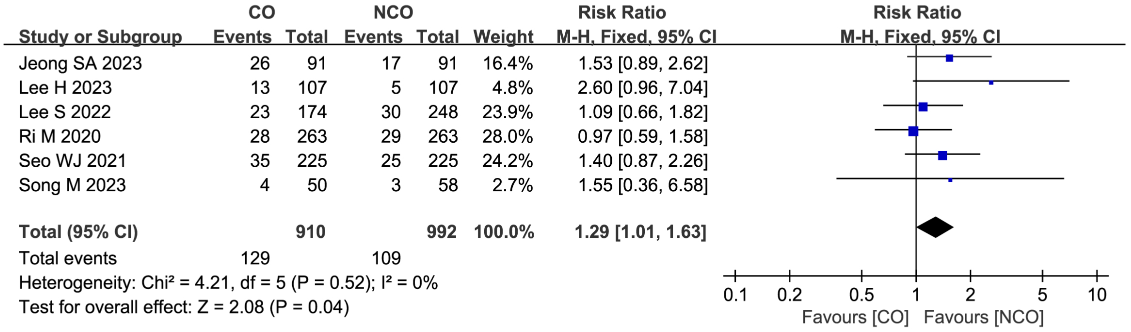

Figure S 20. Forest plot of the peritoneal recurrence rate in subgroup analysis2. [6, 7, 11, 25-27]
